# Enhancing gut microbiota and microbial function with inulin supplementation in children with obesity

**DOI:** 10.1038/s41366-024-01590-8

**Published:** 2024-07-20

**Authors:** Chonnikant Visuthranukul, Sira Sriswasdi, Surapun Tepaamorndech, Supakarn Chamni, Asada Leelahavanichkul, Yutthana Joyjinda, Vitavat Aksornkitti, Sirinuch Chomtho

**Affiliations:** 1 Center of Excellence in Pediatric Nutrition, Division of Nutrition, Department of Pediatrics, Faculty of Medicine, Chulalongkorn University, Bangkok, 10330 Thailand; 2 Research Affairs, Faculty of Medicine, Chulalongkorn University, Bangkok, 10330 Thailand; 3 Center of Excellence in Computational Molecular Biology, Faculty of Medicine, Chulalongkorn University, Bangkok, 10330 Thailand; 4 Department of Microbiology, Faculty of Medicine, Chulalongkorn University, Bangkok, 10330 Thailand; 5 Center of Excellent in Natural Products and Nanoparticles (NP2), Department of Pharmacognosy and Pharmaceutical Botany, Faculty of Pharmaceutical Sciences, Chulalongkorn University, Bangkok, 10330 Thailand; 6 Center of Excellence in Inflammation and Immunology Research Unit (CETRII), Department of Microbiology, Faculty of Medicine, Chulalongkorn University, Bangkok, 10330 Thailand; 7 WHO-CC for Research and Training on Viral Zoonoses, Faculty of Medicine, Chulalongkorn University, Bangkok, 10330 Thailand; 8 Thai Red Cross Emerging Infection Diseases-Health Science Center, Bangkok, 10330 Thailand; 9 Department of Anatomy, Faculty of Medicine, Chulalongkorn University, Bangkok, 10330 Thailand

**Keywords:** Randomized controlled trials, Obesity, Paediatrics

## Abstract

**Background and objectives:**

Gut dysbiosis that resulted from the alteration between host-microbe interaction might worsen obesity-induced systemic inflammation. Gut microbiota manipulation by supplementation of prebiotic inulin may reverse metabolic abnormalities and improve obesity. This study aimed to determine whether inulin supplementation improved intestinal microbiota and microbial functional pathways in children with obesity.

**Methods:**

Children with obesity whose BMI above median + 2SDs were recruited to a randomized, double-blinded placebo-controlled study. The participants aged 7–15 years were assigned to inulin supplement extracted from Thai Jerusalem artichoke (intervention), maltodextrin (placebo), and dietary fiber advice groups. All participants received similar monthly conventional advice and follow-up for 6 months. Fecal samples were collected for gut microbiome analysis using 16S rRNA sequencing. Phylogenetic Investigation of Communities by Reconstruction of Unobserved States was performed to infer microbial functional pathways.

**Results:**

One hundred and forty-three children with available taxonomic and functional pathway abundance profiles were evaluated. A significant increase in alpha-diversity was observed in the inulin group. Inulin supplementation substantially enhanced *Bifidobacterium*, *Blautia*, *Megasphaera*, and several butyrate-producing bacteria, including *Agathobacter*, *Eubacterium coprostanoligenes*, and *Subdoligranulum*, compared to the other groups. The inulin group showed a significant difference in functional pathways of proteasome and riboflavin metabolism. These changes correlated with clinical and metabolic outcomes exclusively in the inulin group.

**Conclusions:**

Inulin supplementation significantly promoted gut bacterial diversity and improved gut microbiota dysbiosis in children with obesity. The modulation of functional pathways by inulin suggests its potential to establish beneficial interactions between the gut microbiota and host physiology. Inulin supplementation could be a strategic treatment to restore the balance of intestinal microbiota and regulate their functions in childhood obesity.

## Introduction

Obesity is a global health concern and the number of children as well as adolescents with overweight or obesity is dramatically high [1]. These children are prone to develop several co-morbidities such as dyslipidemia, non-alcoholic fatty liver disease, and type 2 diabetes mellitus [2].

Recent evidence has suggested that the gut microbiota is involved in energy regulation along with an inflammatory status [3]. The imbalance or dysbiosis of the gut microbiota should be recognized as environmental factors that influence the pathophysiological mechanisms underlying obesity [4]. Many studies in humans have shown differences in the composition and diversity of gut microbiota between individuals with and without obesity [5–7]. Obesity in humans has already been associated with the low relative abundance of Bacteroidetes and Actinobacteria, but high relative abundance of Firmicutes [5, 8]. Therefore, consumption of prebiotics, utilized by gut microbiota to regulate host physiology, could be a strategic treatment, and reverses metabolic abnormalities in childhood obesity [8]. Prebiotic supplementation promoted an increase in *Bifidobacterium* and other beneficial microbes related to their positive effects on host health via microbiota-derived bioactive molecules, such as short chain fatty acids (SCFAs), leading to decreased body weight and adiposity partly through the attenuation of metabolic derangement [9]. There have been studies regarding the effects of prebiotics on gut microbiota in adults with obesity; however, the outcomes were inconsistent [10, 11]. Furthermore, effects of prebiotics on gut microbiota may differ between adult and pediatric populations. Unfortunately, there have been very few interventional studies about prebiotics in children with obesity. For example, researchers found oligofructose-enriched inulin (OI) increased *Bifidobacterium* by using PCR technique, but not by 16S rRNA sequencing compared to the placebo [12]. There was a study about positive effects of OI on the change in satiety hormones and energy intake [13]. Another study in children and adolescents with obesity showed that oligofructose supplementation for 12 weeks had no effect on body weight in this population [14]. These suggest that studies of the impact of prebiotic supplementation on childhood obesity were very limited and remain inconclusive. Therefore, we aimed to evaluate the effects of prebiotic supplementation as inulin on gut microbiota and microbial functional pathways in children with obesity. Moreover, the relationships between the change in gut microbiota and the changes in clinical or metabolic features were assessed.

## Methods

### Study participants

This study was a randomized double-blinded placebo-controlled trial conducted from August 2017 to July 2020 at the King Chulalongkorn Memorial Hospital (KCMH), Thailand. The detailed protocol was previously described [15]. This trial was registered at clinicaltrials.gov as NCT03968003. Children with obesity aged 7–15 years whose body mass index (BMI) were above median plus 2 standard deviations (SDs) from the WHO growth reference [1] were enrolled from the KCMH and the social media. All the children who met the inclusion criteria were recruited as mentioned in the previously published study [15].

### Study design

The detailed study design has been mentioned elsewhere [15]. In brief, one hundred and sixty-five participants were randomly assigned to three groups. In the inulin group, participants consumed the extracted inulin powder from Thai Jerusalem artichoke, using our patented technique, approximately 30 min before dinner each day. Similarly, the placebo group received isocaloric maltodextrin using the same procedure. The third group received structured dietary fiber advice, including portion size illustrations, to ensure appropriate dietary fiber intake for their age [16, 17]. All participants obtained the same conventional advice about diet, exercise, and behavior modification and monthly follow-up for 6 months.

### Assessment of dietary intake, physical activity, anthropometry, body composition, and metabolic profiles

The details have been published elsewhere [15]. In brief, dietary intake was evaluated by a dietician using 3-day dietary records. The daily energy and nutrients intake were calculated using the Institute of Nutrition, Mahidol University Calculation-Nutrients (INMUCALs) Version 3 [18]. Physical activity was assessed by questionnaires. Anthropometry was collected by trained personnel and BMI Z-scores were calculated based on WHO 2007 growth reference using the WHO Anthroplus program [19]. Body composition was measured by bioelectrical impedance analysis using the InBody 770 (InBody Co., Ltd., Chungcheongnam-do, KOREA) and then fat mass index (FMI), and fat-free mass index (FFMI) were calculated [20]. For metabolic profiles, venous blood was attained after a 12-h fast to evaluate lipid profiles and alanine aminotransferase (ALT) which were determined according to the methods in the previous study [15].

### Fecal collection, DNA isolation, and 16S PCR amplification

Fresh fecal samples were collected at baseline, the 3^rd^, and 6^th^ month visits of the study for gut microbiota and SCFAs analyses. The details have been published elsewhere [5]. In brief, participants were provided with sterile stool collection kits, containers, and temperature-controlled packages and instructed to collect fecal samples at home. Each participant placed a fresh stool sample into one half of a 50 ml sterile container, which was then double-sealed in a zip-lock bag. The samples were stored in the freezer compartment of a home refrigerator (approximately −20 °C) and delivered to the laboratory within 24 h. Upon arrival, the samples were stored at −80 °C until analysis.

Fecal samples were resuspended in InhibitEX buffer (QIAGEN, Germany), incubated at 70 °C for 5 min, and centrifuged at 20,000 × *g* for 1 min. Supernatant was collected for DNA isolation by using QIAamp fast DNA stool mini kit (QIAGEN, Germany) in accordance with the manufacturer’s instructions and 16S PCR amplification was mentioned in the previous published study [5].

### 16S rRNA sequencing processing and analysis

Illumina paired-end reads of 16S rRNA from participants collected from the baseline, 3^rd^, and 6^th^ month visits were analyzed as previously described [5]. Briefly, the quality of the data were inspected using FastQC [21]. All reads were processed by QIIME2 (v.2020.8) [22] and clustered at a similarity threshold of 97% to define operational taxonomic units (OTUs). Potential chimeric reads were removed using the *q2-dada2* plugin [23] with default parameters. Taxonomic annotations were performed using the *q2-feature-classifier* plugin with the *classify-sklearn* option and confidence cutoff of 0.7. This method implements a Naïve Bayes classifier that was trained on the non-redundant SILVA 16s rRNA database (v.132) [24, 25]. Unclassified sequences were labeled as “unknown”.

Microbial functions and pathway abundances, based on the Kyoto Encyclopedia of Genes and Genomes (KEGG) ontology, were annotated using the Phylogenetic Investigation of Communities by Reconstruction of Unobserved States 2 (PICRUSt 2) software [26]. The abundance of each pathway was estimated based on the total copy number of genes involved in that pathway and the abundance of microbial taxa possessing those genes.

### Differential analysis of relative taxonomic and KEGG pathway abundances

To normalize for different read depths across samples, abundance values were converted to percentages. Only genera and KEGG pathways that were detected (abundance >0) in at least 20% of the samples were retained. Overall, a total of 104 genera and 147 KEGG pathways were considered. The differences in abundance percentages between the 6^th^ month and baseline were calculated for each participant and compared across the three groups using non-parametric tests (Mann–Whitney *U* test and Wilcoxon signed rank test). Alpha-diversity was measured using Shannon entropy and beta-diversity was calculated using Bray–Curtis dissimilarity.

### Co-occurrence network analysis for KEGG pathways

To identify more important KEGG pathways beyond those whose abundances changed across treatment groups, we examined interactions between pathways through their co-occurrences. Pathways that significantly co-occurred with others (absolute Spearman’s correlation coefficient > 0.5 and adjusted *p* values < 0.05) were selected to construct a co-occurrence network. The betweenness centrality score was calculated using the *networkx* Python library and normalized to a scale of 0.0–1.0. Pathways with high normalized betweenness (> 0.5) were considered important in the context of inter-pathway interactions. For visualization, pathways whose abundances substantially changed between the 6^th^ month and baseline (unadjusted Mann–Whitney *U* test *p* value < 0.05) were selected.

### Short chain fatty acids

Fecal samples were prepared for analysis of SCFAs by diluting 10-fold with phosphate-buffered saline (pH 8.0) using a stomacher blender (Stomacher^®^ 80 Biomaster; Seward, Worthing, UK) for 5 min as stated in the method of Kisuse et al. [27]. Then a 1 ml of fecal slurry was centrifuged at 13,000 × *g* for 5 min, and the supernatant was stored at −80 °C.

Lactic acid and SCFAs, including acetic acid, butyric acid, and propionic acid, from the fecal samples were investigated by high-performance liquid chromatography (Water 1525, USA). The samples were prepared by the method as previously defined with some modifications [28, 29]. All parameters were evaluated by Agilent Technologies 7890A equipment (Santa Clara, USA).

### Statistical analysis

All statistical tests were performed using Python programming language (v.3.10.12). Differential abundance analyses with Mann–Whitney *U* tests and Wilcoxon signed rank tests were performed using the *mannwhitneyu* and the *wilcoxon* function from the *scipy* library. Spearman’s correlation coefficients were calculated using the *spearmanr* function from the *scipy* library, which also reported *p* values. Benjamini–Hochberg corrections were performed and an adjusted *p* value cutoff of 0.05 was applied to define statistical significance. Associations between changes in taxonomic or pathway abundances and changes in clinical features were measured using Pearson’s correlation coefficients.

## Results

A total of 165 Thai children with obesity participated in the study (mean age: 10.4 ± 2.2 years, 59% male). They were randomly allocated to the placebo, inulin, and dietary fiber advice groups. Only 143 participants who completed the study with available taxonomic and functional pathway abundance profiles at the baseline, 3^rd^, and 6^th^ month visits were included in this study. There was no difference in the attrition rate between the three groups. Neither participants receiving inulin nor those receiving placebo had any significant side effects [15].

### Baseline characteristics and gut microbiota

Demographic data and baseline characteristics of all groups were illustrated elsewhere [15]. There were no significant differences in baseline anthropometry, clinical data, nutrient intake, physical activity, or biochemical markers. Moreover, baseline gut microbiota diversity and composition were not significantly different among the three groups (*p* > 0.05) (Table 1).

**Table 1:** Baseline microbial abundance at the phylum and genus levels in children with obesity.

Taxonomic group^a^	Mean microbial abundance *Z*-score ± SD		
	Placebo (*n* = 46)	Inulin (*n* = 48)	Dietary fiber advice (*n* = 49)
Phylum			
Actinobacteria	2.380 ± 4.814	2.647 ± 5.979	2.408 ± 3.852
Bacteroidetes	38.013 ± 27.079	34.160 ± 28.307	29.936 ± 26.124
Firmicutes	42.973 ± 21.497	44.731 ± 24.552	52.552 ± 24.137
Fusobacteria	1.056 ± 2.480	1.661 ± 4.412	0.540 ± 1.970
Proteobacteria	14.157 ± 17.950	16.047 ± 19.974	13.739 ± 16.334
Genus			
*Agathobacter*	2.529 ± 4.801	1.125 ± 2.279	3.037 ± 4.560
*Akkermansia*	0.818 ± 3.540	0.228 ± 0.849	0.380 ± 1.095
*Alistipes*	3.246 ± 5.002	1.982 ± 3.841	2.343 ± 2.761
*Anaerostipes*	1.018 ± 2.257	0.615 ± 1.460	0.645 ± 1.033
*Bacteroides*	23.680 ± 21.060	24.585 ± 25.001	14.246 ± 16.526
*Barnesiella*	1.052 ± 1.904	0.392 ± 0.739	1.052 ± 1.904
*Bifidobacterium*	1.398 ± 2.463	1.812 ± 4.484	1.962 ± 3.780
*Blautia*	3.643 ± 7.401	2.850 ± 7.147	3.450 ± 3.960
*Clostridium_sensu_stricto_1*	0.553 ± 1.617	0.654 ± 1.892	1.184 ± 3.719
*Collinsella*	0.945 ± 2.525	0.759 ± 3.289	0.370 ± 0.489
*Dialister*	1.400 ± 3.933	1.833 ± 3.932	1.274 ± 2.855
*Dorea*	1.053 ± 1.873	0.649 ± 1.296	0.957 ± 1.688
*Enterobacter*	0.706 ± 3.503	0.814 ± 2.046	0.583 ± 1.350
*Escherichia-Shigella*	6.635 ± 13.330	6.845 ± 12.880	4.161 ± 7.479
*Eubacterium coprostanoligenes*	1.081 ± 1.894	0.651 ± 1.762	1.697 ± 3.245
*Faecalibacterium*	3.972 ± 4.126	5.773 ± 9.480	7.214 ± 7.818
*Fusicatenibacter*	0.598 ± 0.864	0.680 ± 1.727	0.909 ± 2.122
*Fusobacterium*	1.041 ± 2.486	1.477 ± 4.291	0.540 ± 1.970
*Incertae_Sedis*	0.347 ± 0.636	0.280 ± 0.548	0.978 ± 3.548
*Klebsiella*	2.942 ± 6.378	1.804 ± 3.319	2.255 ± 4.595
*Lachnospiraceae_NK4A136_group*	0.901 ± 2.300	1.981 ± 5.573	1.122 ± 1.804
*Megamonas*	1.442 ± 7.508	1.263 ± 5.484	0.390 ± 0.832
*Megasphaera*	2.163 ± 4.867	5.511 ± 13.830	2.719 ± 6.947
*Parabacteroides*	1.152 ± 1.119	0.929 ± 1.233	0.747 ± 0.944
*Parasutterella*	0.331 ± 0.674	1.835 ± 6.709	0.600 ± 1.290
*Phascolarctobacterium*	2.751 ± 4.237	3.035 ± 5.613	3.756 ± 5.584
*Prevotella*	7.851 ± 15.166	5.144 ± 11.452	10.592 ± 19.206
*Romboutsia*	0.757 ± 1.572	1.982 ± 5.077	1.497 ± 3.885
*Ruminococcus gnavus*	0.868 ± 2.622	0.451 ± 1.067	0.579 ± 1.022
*Ruminococcus torques*	1.612 ± 3.038	1.123 ± 2.487	1.482 ± 4.934
*Roseburia*	2.464 ± 3.018	1.514 ± 1.829	2.048 ± 2.049
*Subdoligranulum*	1.764 ± 2.452	1.915 ± 4.259	2.201 ± 3.907
*Sutterella*	0.871 ± 1.806	0.951 ± 2.874	0.750 ± 1.461
*UCG-002*	0.878 ± 1.728	0.689 ± 1.307	1.098 ± 1.950
*Veillonella*	0.422 ± 1.017	0.707 ± 1.175	0.921 ± 2.019

^a^The taxonomy group of the top 5 most abundant phyla and top 35 most abundant genera in the three groups was shown in the table.

### Effects on gut microbiota diversity and composition

**Fig. 1:**
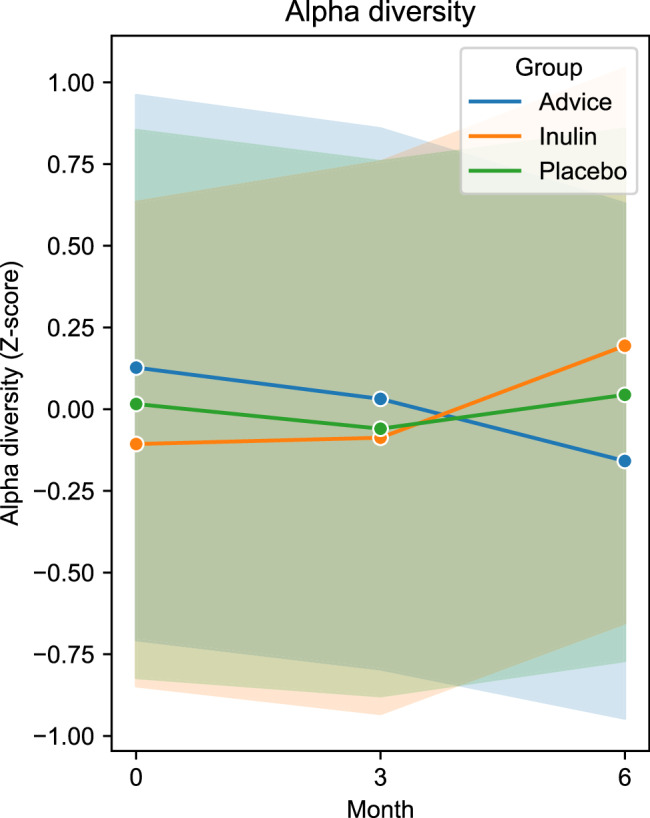
Change of alpha-diversity (Shannon Index) at the genus level in the placebo, inulin, and dietary fiber advice groups over time (month 0, 3, and 6). Between group analysis was performed by Mann–Whitney *U* test.

Based on community-wide analysis, there were 15 phyla, 118 families, and 340 genera detected in our study. There was no significant difference in beta-diversity among the three groups at the phylum level and the genus level (Supplementary Fig. 1). Alpha-diversity (Shannon Index), the index assessing the variety of intestinal microbiota, was not significantly different at the phylum level. However, the alpha-diversity greatly increased in the inulin group compared to the placebo and dietary fiber advice groups at the genus level (*p* = 0.028 and *p* = 0.026) (Fig. 1). In addition, the alpha-diversity significantly increased from baseline to the 6^th^ month in the inulin group (*p* = 0.033). The other groups exhibited no significant change.

The changes of microbial abundance at the phylum level from month 0 and month 6 were shown in Supplementary Fig. 2. There were no significant differences in the change of relative abundance of Actinobacteria (Supplementary Fig. 2A), Bacteroidetes (Supplementary Fig. 2B), Firmicutes (Supplementary Fig. 2C), Fusobacteria, and Proteobacteria (data not shown) among the placebo, inulin, and dietary fiber advice groups.

**Fig. 2:**
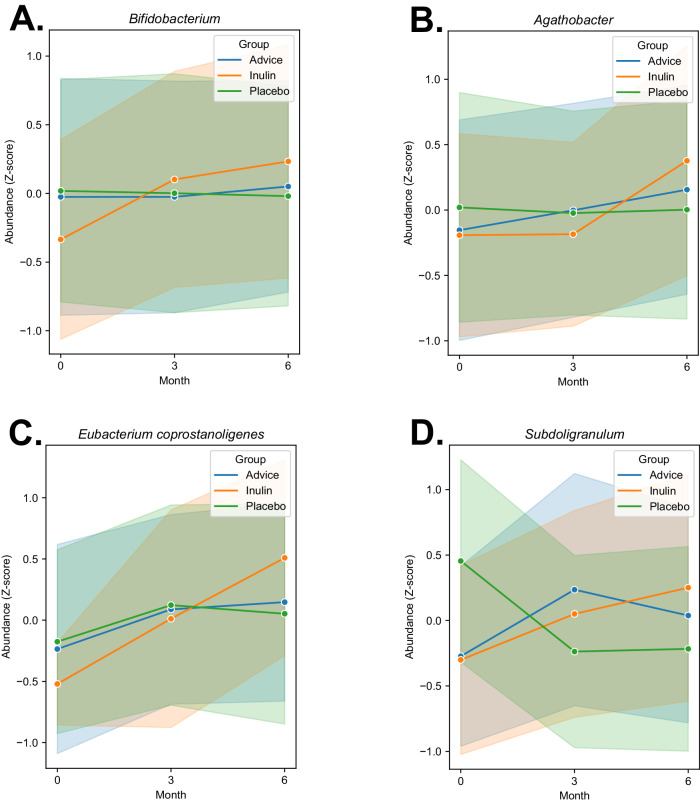
The changes in relative abundances at the genus level in children with obesity between the three groups. **A** *Bifidobacterium*, **B** *Agathobacter,* **C** *Eubacterium* *coprostanoligenes,* and **D** *Subdoligranulum*. The relative abundances were processed by the QIIME2 platform. Between group analysis was performed by Mann-Whitney U test.

However, at the genus level, a substantial elevation in the relative abundance of *Bifidobacterium* was found after 6-month inulin supplementation compared to the placebo and dietary fiber advice groups (*p* = 0.0058 and *p* = 0.025, respectively) (Fig. 2A). Notably, the results showed a significant enrichment of butyrate-producing bacteria, *Agathobacter*, in the inulin group compared to the placebo group (*p* = 0.04), and this genus had a tendency of elevation compared to the dietary fiber advice group (*p* = 0.05) (Fig. 2B). Inulin supplementation greatly enhanced *Eubacterium coprostanoligenes* compared to the placebo group (*p* = 0.04) (Fig. 2C). A significant increase in the relative abundance of the other bacteria in the group of butyrate-producing bacteria, *Subdoligranulum*, was found after the 6-month inulin supplementation compared to the placebo group (*p* = 0.009) (Fig. 2D). The dietary fiber advice group also showed an increase in *Subdoligranulum* compared to the placebo group (*p* = 0.04). *Blautia* was greatly enhanced after inulin supplementation compared to the dietary fiber advice group (*p* = 0.03). *Megasphaera* was significantly increased in the inulin group compared to the placebo group (*p* = 0.03) and had a tendency of elevation compared to the dietary fiber advice group (*p* = 0.088).

For within group analysis, *Bifidobacterium* was significantly increased from baseline to the 6^th^ month in the inulin group (*p* = 0.0025), but this result was not observed in the placebo and dietary fiber advice groups. Within-group analysis showed significant differences in the relative abundance of *Agathobacter, Eubacterium coprostanoligenes, and Subdoligranulum* in the inulin group (*p* = 0.016, *p* = 0.04, and *p* = 0.049, respectively). While there was no significant difference in relative abundance of these SCFA-producing bacteria from baseline to the 6^th^ month in the other groups. *Blautia* was significantly increased in the inulin group after the 6-month study period, whereas no significant difference of this genus was found in the other groups. Moreover, the levels of *Megasphaera* tended to increase from baseline to the 6^th^ visit only in the inulin group (*p* = 0.08). The microbial metabolites, fecal SCFAs, tended to increase in all groups, whereas no significant difference between groups was observed (data not shown).

### Changes in microbial functions

**Fig. 3:**
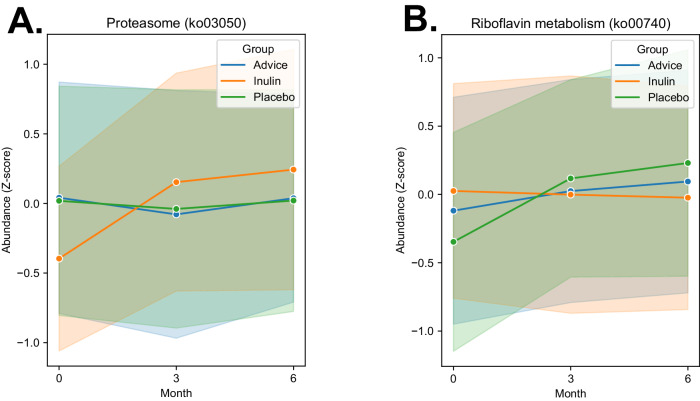
The changes in the impacted functional pathways in children with obesity between the three groups. **A** The pathway of proteasome (ko03050) and **B** The riboflavin metabolism (ko00740). Between group analysis was perform by Mann–Whitney *U* test.

The change of microbial functions between groups after the 6-month study period was assessed. The proteasome pathway (ko03050) was greatly upregulated in the inulin group compared to the placebo and dietary fiber advice groups (*p* = 0.017 and *p* = 0.028, respectively) (Fig. 3A). The riboflavin metabolism pathway (ko00740) significantly increased in the placebo group compared to the inulin group (*p* = 0.027) (Fig. 3B). Within group analysis of the changes in pathway abundances, demonstrated as co-occurrence networks (Supplementary Fig. 3A–C), showed that different pathways were impacted among the three groups. For instance, the proteosome pathway (ko03050) and the ribosome biogenesis pathway (ko03008) were substantially increased only in the inulin group while the histidine metabolism pathway (ko00340) was significantly elevated in the placebo and dietary fiber advice groups.

**Fig. 4:**
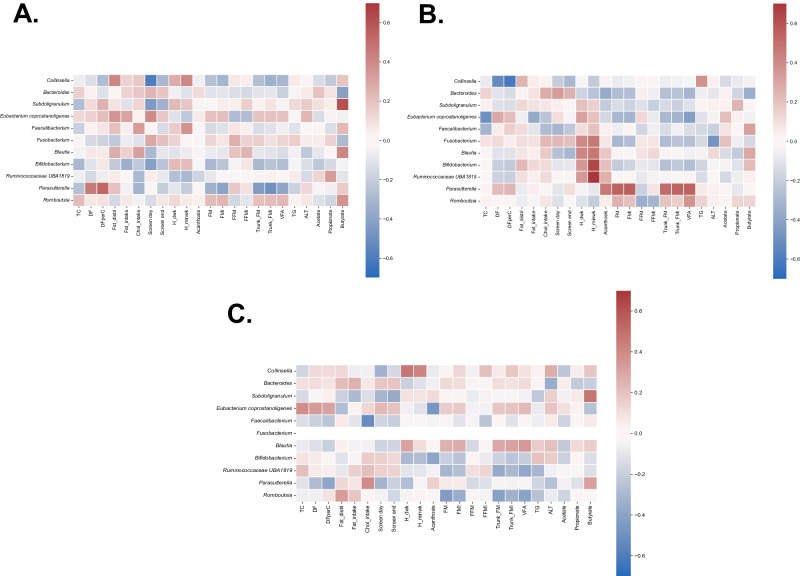
Heatmap of the correlation analysis between the changes of gut microbiota and clinical as well as metabolic features in 6 months. **A** Placebo group, **B** Inulin group, and **C** Dietary fiber advice group. ALT alanine aminotransferase, Chol_intake cholesterol intake, DF dietary fiber intake, DFperC dietary fiber intake, g per 1000 kcal, Fat_distri fat distribution, FFM fat-free mass, FFMI fat-free mass index, FM fat mass, FMI fat mass index, H_dwk high intensity exercise, days per week, H_minwk high intensity exercise, minutes per week, Screen day screen time on weekday, Screen end screen time on weekend, TC total calories, TG triglyceride, VFA visceral fat area.

The relationship between the changes in gut microbiota and the changes in clinical and metabolic features after the 6-month period was notable in the inulin group (Fig. 4B) compared to the placebo and dietary fiber advice groups (Fig. 4A, C). Regarding the inulin group, *Faecalibacterium* abundance change positively correlated with the change of butyrate. The change in *Fusobacterium* abundance was negatively associated with ALT. *Parasutterella* abundance change was positively correlated with the change in adiposity, including FM, FMI, trunk FM, trunk FMI, and visceral fat area (VFA). The change in *Romboutsia* abundance was negatively correlated with FFM and FFMI, but positively correlated with VFA. The change in *Eubacterium coprostanoligenes group* was negatively correlated with high energy intake. The change in *Subdoligranulum* abundance was negatively associated with BMI *Z*-score. However, these relationship patterns were completely unobserved in the placebo and dietary fiber advice groups.

## Discussion

As far as we know, this is the largest randomized control trial (RCT) study with gut microbiota and metabolomic evaluations of a prebiotic intervention in children with obesity. Here, inulin enhanced several SCFA-producing bacteria, especially *Bifidobacterium* spp., and increased proteasome pathway with decreased riboflavin metabolism (microbial function analysis) when compared to the control. As such, obesity induced gut dysbiosis [30, 31] that was attenuated by inulin as indicated by the increased variety of gut microbiota (alpha-diversity) after the 6-month intervention. Although a previous RCT using OI did not demonstrate alterations in alpha-diversity, the elevation of *Bifidobacterium* spp. [12] was mentioned similar to the use of other prebiotics [10, 11]. Additionally, several inulin-induced bacteria as we found were SCFA-producing bacteria with the well-known benefits against obesity. Accordingly, *Agathobacter* was found in adults with obesity achieving weight control after 12-week high fiber rye administration [32] and in patients receiving oatmeal [33]. *Eubacterium coprostanoligenes* was found in the healthy individuals [34] with an essential role of SCFA production [35], and *Subdoligranulum* regulates host energy and reduced inflammation [36] with negatively associated with adipocyte and other metabolic disturbances [37]. *Blautia* was found primarily in the lean individuals [38] and the well-controlled type 2 diabetes cases [39], while *Megasphaera* was elevated in the individuals with obesity after treatment [40]. Based on clinical results from our previously published study, we found a significant increase in fat-free mass along with enhancement of *Bifidobacterium* and several SCFA-producing bacteria only in the inulin group. Optimizing the intestinal microbial ecosystem has been linked to reduced inflammation and increased anti-inflammatory activity [12]. *Bifidobacterium* metabolizes inulin to produce substrates necessary for other SCFA-producing bacteria, thus facilitating SCFA synthesis [41]. Taken together, metabolites derived from these bacteria would manifest anti-inflammatory activity, then possibly promoting myogenesis [42] as we found fat-free mass gain. Further study of gut-muscle axis response from inulin supplementation in childhood obesity is warranted. Despite the possible influences of SCFAs in obesity, our results demonstrated a similar pattern of fecal SCFA profiles among the three groups which might be because these metabolites were quickly absorbed into the body, making it difficult to detect the difference in feces.

Interestingly, the anti-inflammatory SCFAs might attenuate obesity-induced chronic inflammation that is partly modulated through inflammasome pathway [43, 44], especially nuclear factor-κB (NF-κB) and activator protein 1 [45, 46]. Because proteasomes partly regulate NF-κB pathway [47], modulation of proteasome-dependent NF-κB; for example, through inulin supplement, might conserve gut homeostasis. Conversely, riboflavin regulates the pro-inflammatory activity of macrophages and plays a key role in mediating obesity-related inflammation [48, 49]. Its metabolism significantly increased in the placebo group compared to the inulin group, indicating that inulin might attenuate inflammation resulting in less riboflavin metabolism for inflammatory process.

Additionally, within group analysis of functional changes in the inulin group showed a different pattern of significant pathways compared to the other groups. Proteasome pathway significantly increased only in the inulin group, consistent with between-group analysis. Stazar et al. found that histidine metabolism positively affects tumor necrosis factor-α stimulation in African participants [50]. In our study, this pathway significantly increased in the placebo and dietary fiber advice groups, but remained unchanged in the inulin group, suggesting that inulin might mitigate inflammation. The ribosome biogenesis plays an essential role in protein synthesis, leading to regulation of skeletal muscle growth and muscle building. This finding agreed with the result of fat-free mass gain in our previous RCT study [15].

Notably, we found several relationships between the changes in gut microbiota and the changes in clinical and metabolic features exclusively in the inulin group. For example, high relative abundance of *Parasutterella* involved in systemic low-grade inflammation and activation of the fatty acid biosynthesis pathway leading to weight gain [51], as we found this genus had a positive direction to adiposity. We observed a positive relationship between *Romboutsia* and adiposity, consistent with previous studies linking *Romboutsia* to obesity [52]. *Eubacterium coprostanoligenes group* revealed a negative relationship with body weight and cholesterol in animal models [53] and a longitudinal study demonstrated that *Subdoligranulum* was negatively associated with future BMI in childhood [54]. The findings of both genera were relevant to our results. All these relationship patterns in the inulin group suggested that inulin supplementation might establish favorable connections of gut microbiota with clinical features and biochemical markers.

### Strengths and limitations of the study

This present study appears to be the largest RCT documenting the change of gut microbiota and microbial functions after prebiotic supplementation in children with obesity. The study indicated that the supplementation of the extracted inulin from Thai Jerusalem artichoke showed an effect on enrichment of favorable microbes with a novel finding of an increased alpha-diversity in children with obesity. We used next-generation sequencing technology, the 16S rRNA sequencing, to analyze the bacterial sequences, which was a ubiquitous technique to identify bacterial community in the intestine. The limitation of this study was the assessment of fecal SCFAs which might be difficult to detect any difference between groups. However, the results of fecal SCFAs might not explain an entire process of metabolic changes. Further exploration of serum SCFAs could lead to better understanding of host energy harvest and storage mechanism.

## Conclusions

Inulin supplementation could be a strategic treatment to selectively promote beneficial microbes and restore the balance of intestinal microbiota in children with obesity. Moreover, the predominantly functional characteristics of the gut microbiota and their associations with clinical and metabolic parameters could be attributed to inulin supplementation, leading to different patterns from the placebo and dietary fiber advice groups. Further exploration of the impacts of inulin supplementation as a long-term intervention on gut microbiota and their metabolites could elicit deeper knowledge of host-microbe interaction in pediatric obesity.

## Supplementary information

Supplement Figure 1, 2, 3 and Legend

Reporting checklist

CONSORT

## Data Availability

Data described in the manuscript will be made available upon request pending application and approval from the corresponding author. 16S rRNA sequencing data (FASTQ files) were submitted to the Sequence Read Archive (SRA) under the reference PRJNA1096061.
