# Supplementary material for: Enhancing gut microbiota and microbial function with inulin supplementation in children with obesity
Source: Int J Obes (Lond). 2024 Jul 20;48(12):1696–704. doi: 10.1038/s41366-024-01590-8 (PMC11584386; doi:10.1038/s41366-024-01590-8)
Supplement: Supplementary file 1 — Supplement Figure 1, 2, 3 and Legend [file 41366_2024_1590_MOESM1_ESM.pdf]

Supplement Figure 1 Change of beta-diversity at the genus level in the placebo, inulin, and dietary fiber advice groups over time at the baseline (month 0) and the end of the study (month 6). Bray-Curtis was used for data comparisons.

Supplement Figure 2 Change of relative abundance of (A) Actinobacteria, (B) Bacteroidetes, and (C) Firmicutes at the genus level in the placebo, inulin, and dietary fiber advice groups over time (month 0, 3, and 6). The relative abundances were processed by the QIIME2 platform. Between group analysis was performed by Mann-Whitney U test.

Supplement Figure 3 The within-group co-occurrence network of KEGG functional pathways for (A) the placebo group, (B) the inulin group, and (C) the dietary fiber advice group. Pathways whose abundance changed between month 0 and month 6 in each group (unadjusted P-values < 0.05) were shown in green. Significant pathways (adjusted P-values < 0.05) were highlighted in orange. Blue lines indicate positive co-occurrence with Pearson's correlation > 0.5. Red lines indicate negative co-occurrence with Pearson's correlation < -0.5.

KEGG, Kyoto Encyclopedia of Genes and Genomes

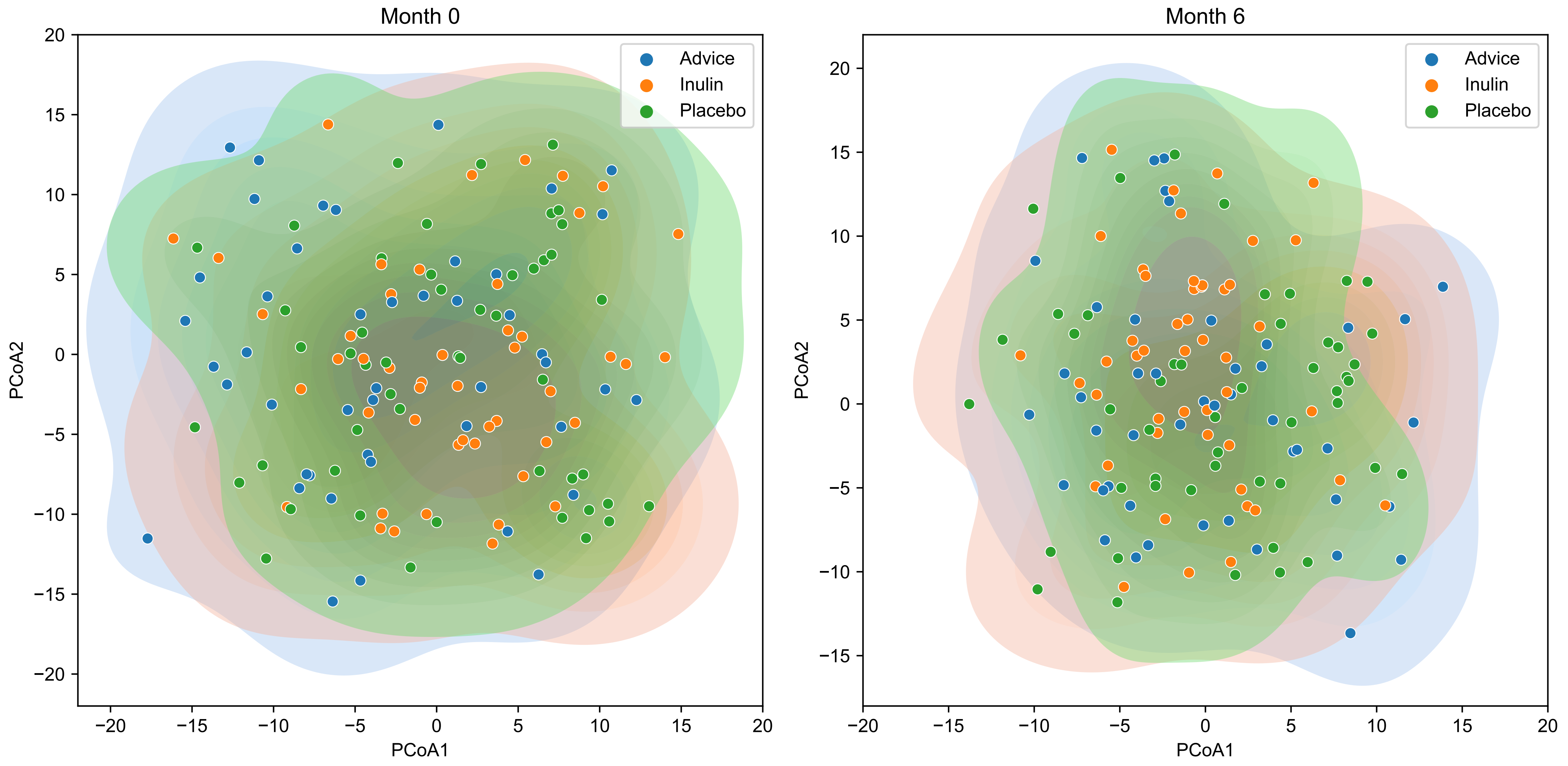
Month 0
Advice
Inulin
Placebo
PCoA2
PCoA1
20
15
10
5
0
−5
−10
−15
−20
Month 6
Advice
Inulin
Placebo
PCoA2
PCoA1

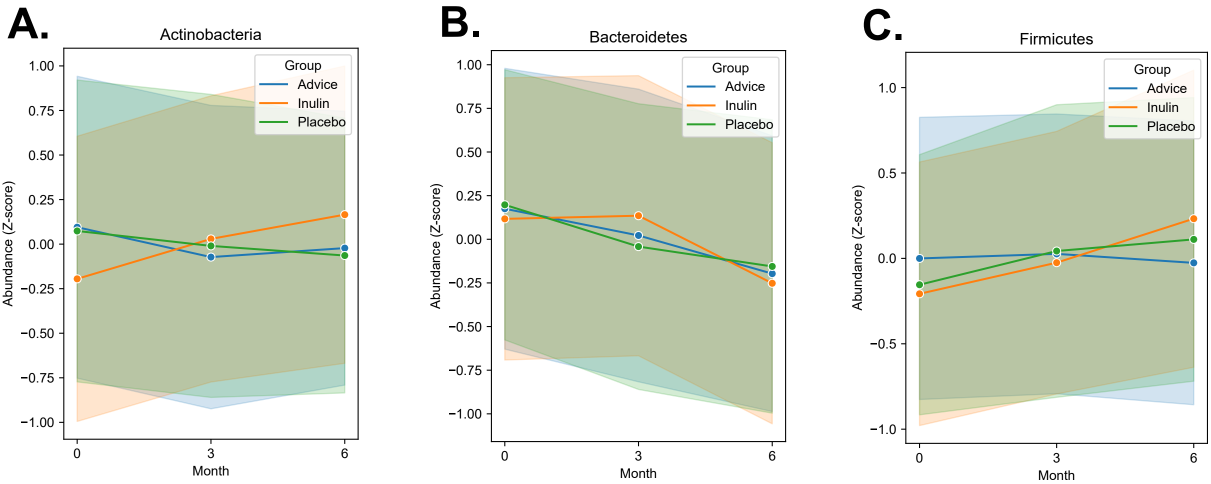

A.
Actinobacteria
B.
Bacteroidetes
C.
Firmicutes
Group
Advice
Inulin
Placebo
Abundance (Z-score)
Month
1.00
0.75
0.50
0.25
0.00
−0.25
−0.50
−0.75
−1.00
1.0
0.5
0.0
−0.5
−1.0
0
3
6

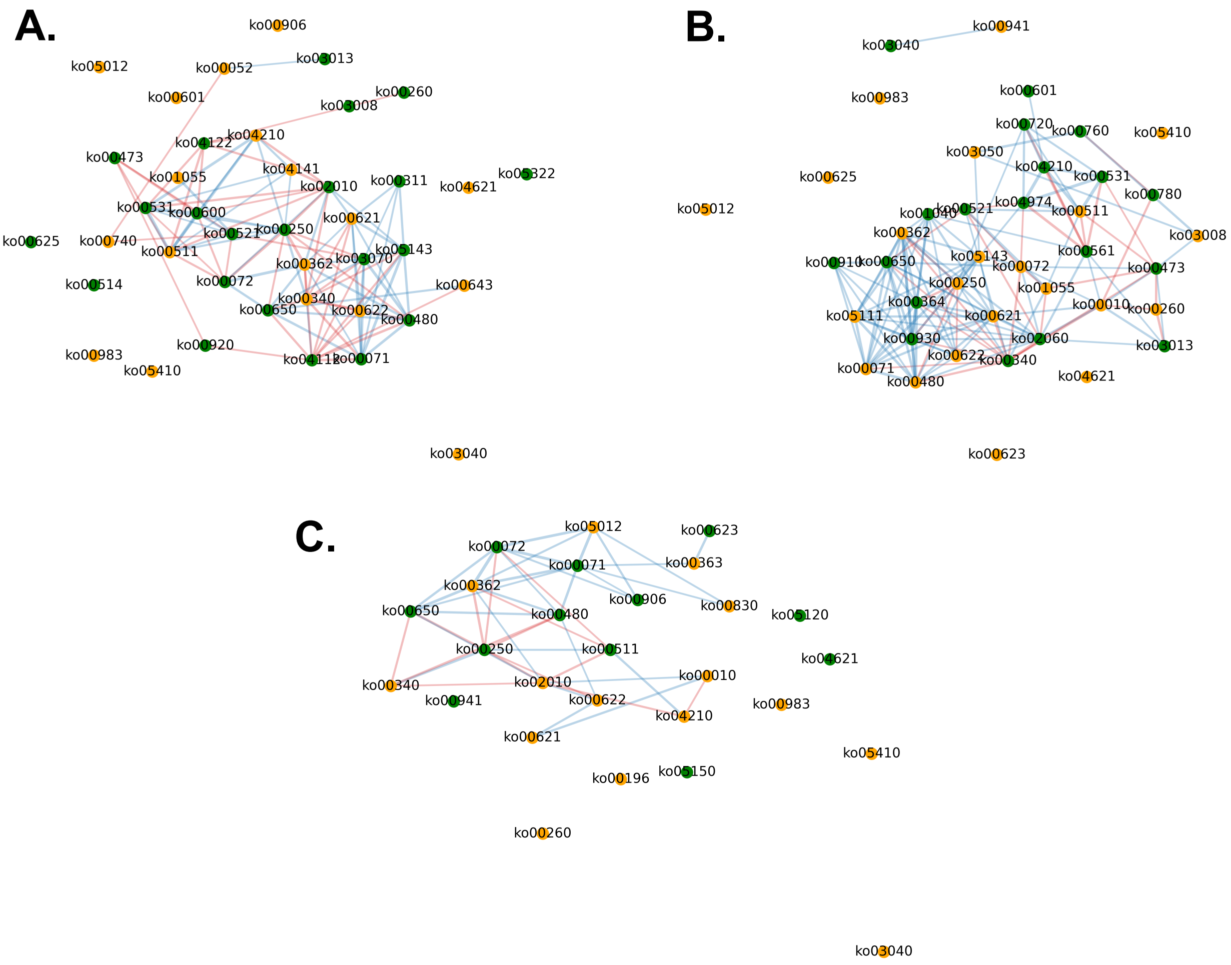
A.
ko00906
ko05012
ko00052
ko03013
ko00601
ko00260
ko03008
ko04210
ko04122
ko00473
ko04141
ko01055
ko00311
ko05322
ko04621
ko02010
ko00531
ko00600
ko00621
ko00250
ko00625
ko00740
ko00521
ko05143
ko00511
ko03070
ko00362
ko00514
ko00072
ko00643
ko00340
ko00650
ko00622
ko00480
ko00920
ko00983
ko04112
ko00071
ko05410
ko03040
B.
ko00941
ko03040
ko00983
ko00601
ko00720
ko00760
ko05410
ko03050
ko04210
ko00625
ko00531
ko00780
ko05012
ko04974
ko00521
ko01040
ko00511
ko03008
ko00362
ko05143
ko00561
ko00910
ko00650
ko00072
ko00473
ko00250
ko01055
ko00364
ko00010
ko00260
ko05111
ko00621
ko00930
ko02060
ko03013
ko00622
ko00340
ko00071
ko04621
ko00480
ko00623
C.
ko05012
ko00623
ko00072
ko00071
ko00363
ko00362
ko00906
ko00830
ko00650
ko00480
ko05120
ko00250
ko00511
ko04621
ko00010
ko00340
ko02010
ko00941
ko00622
ko00983
ko04210
ko00621
ko05410
ko00196
ko05150
ko00260
ko03040
